# Genetic diversity and population structure of the primary malaria vector *Anopheles sinensis* (Diptera: Culicidae) in China inferred by *cox*1 gene

**DOI:** 10.1186/s13071-017-2013-z

**Published:** 2017-02-10

**Authors:** Xinyu Feng, Libin Huang, Lin Lin, Manni Yang, Yajun Ma

**Affiliations:** 1 WHO Collaborating Center for Tropical Diseases, Key Laboratory of Parasite and Vector Biology, Ministry of Public Health, National Institute of Parasitic Diseases, Chinese Center for Disease Control and Prevention, Shanghai, 200025 China; 2 Department of Tropical Infectious Disease, Second Military Medical University, Shanghai, 200433 China; 3 Second Military Medical University Press, Shanghai, 200433 China

**Keywords:** *Anopheles sinensis*, Genetic variation, Population structure, *cox*1

## Abstract

**Background:**

*Anopheles sinensis* is a primary vector for *Plasmodium vivax* malaria in most regions of China. A comprehensive understanding of genetic variation and structure of the mosquito would be of benefit to the vector control and in a further attempt to contribute to malaria elimination in China. However, there is only inadequate population genetic data pertaining to *An. sinensis* currently.

**Methods:**

Genetic variations and structure among populations of *An. sinensis* was examined and analyzed based on the nucleotide sequences of a 662 nt variable region of the mitochondrial *cox*1 gene among 15 populations from 20 collection sites in China.

**Results:**

A total of 453 individuals in 15 populations were analyzed. The *cox*1 gene sequences were aligned, and 247 haplotypes were detected, 41 of these shared between populations. The range of haplotype diversity was from 0.709 (Yunnan) to 0.998 (Anhui). The genealogic network showed that the haplotypes were divided into two clusters, cluster I was at a high level of homoplasy, while cluster II included almost all individuals from the Yunnan population. The Yunnan population displayed a significantly high level of genetic differentiation (0.452−0.622) and a restricted gene flow with other populations. The pairwise *F*_ST_ values among other populations were lower. The AMOVA result showed that the percentage of variation within populations (83.83%) was higher than that among populations (16.17%). Mantel test suggested that geographical distance did not significantly contribute to the genetic differentiation (*R* ^2^ = 0.0125, *P* = 0.59). Neutral test and mismatch analysis results showed that the *An. sinensis* population has undergone demographic expansions.

**Conclusions:**

*Anopheles sinensis* populations showed high genetic polymorphism by *cox*1 gene. The weak genetic structure may be a consequence of low genetic differentiation and high gene flow among populations, except the Yunnan samples. The Yunnan population was isolated from the other populations, gene flow limited by geographical distance and barriers. These findings will provide a theoretical basis for vector surveillance and vector control in China.

**Electronic supplementary material:**

The online version of this article (doi:10.1186/s13071-017-2013-z) contains supplementary material, which is available to authorized users.

## Background

*Anopheles sinensis* Wiedemann is an Oriental species with a wide distribution in China [1]. It is the primary malaria vector in plain regions of central China, especially in the paddy planting areas, and it has also been identified as a pathogenic vector for other disease such as *Brugia malayi*, one type of lymphatic filariasis [2, 3]. Despite its disputable malaria vector capacity, *An. sinensis* is still incriminated as a competent vector for *Plasmodium vivax* malaria due to its abundant population size and wide distribution, which have led to occasional local malaria epidemics or outbreaks throughout history [4, 5].

The morbidity of *P. vivax* malaria has dropped down to a historically low level since the Chinese government initiated the National Malaria Elimination Action Plan Programme in 2010 [6]; however, the ecological habitat and distribution of *An. sinensis* has not changed significantly [7, 8]. In addition, the proportion of imported *P. vivax* malaria cases has notably increased in recent years [9, 10], and so increased knowledge on the genetic structure and the divergence among *An. sinensis* populations are essential to lay out effective strategies for vector control and further malarial elimination.

As one important member of the *Hyrcanus* group, *An. sinensis* has been differentiated from its morphologically indistinguishable sibling species (*An. lesteri*, *An. yatsushiroensis*, and so on) by comparison of sequence data from the second internal transcribed spacer (ITS2) region of the ribosomal DNA (rDNA), which also greatly facilitates the subsequent genetics study [11].

To date, *An. sinensis* populations in China exhibit variations in morphology [12], chromosomes [13, 14], ecology [12, 14], vector capacity [15], the mitochondrial DNA (mtDNA) NADH dehydrogenase subunit 5 (*nad*5) [16] and mtDNA control region [17]. Our research team applied random amplified polymorphic DNA (RAPDs) [18] and microsatellite DNA markers [19] to detect the genetic structure of *An. sinensis* samples, the results revealing considerable polymorphism among populations, the genetic divergence however did not correlate with geographical distance. There were two gene pools in *An. sinensis* populations inferred by microsatellites, these structured populations possibly limiting the migration of genes under pressures/selections, such as insecticides and immune genes against malaria [20].

Different molecular markers may demonstrate different genetic structures. The appropriate markers are usually neutral, such as the mitochondrial cytochrome *c* oxidase subunit I (*cox*1) gene, which was used to analyze genetic variation and population structure of the anopheline mosquitoes [21–27]. The *cox*1 gene is slowly evolving compared to other protein-coding mitochondrial genes and is also widely used for reconstructing molecular phylogenies [28]. Although a few Chinese *An. sinensis* population genetic studies have been reported [16, 18, 19], to obtain a more accurate genetic structure of these populations, more molecular markers and more specimens are needed. In the present study, we sought to elucidate the genetic properties and variability of the *An. sinensis* populations collected from almost all distribution regions in China based on the sequences of the *cox*1 gene.

## Methods

### Mosquito collection and identification

**Fig. 1:**
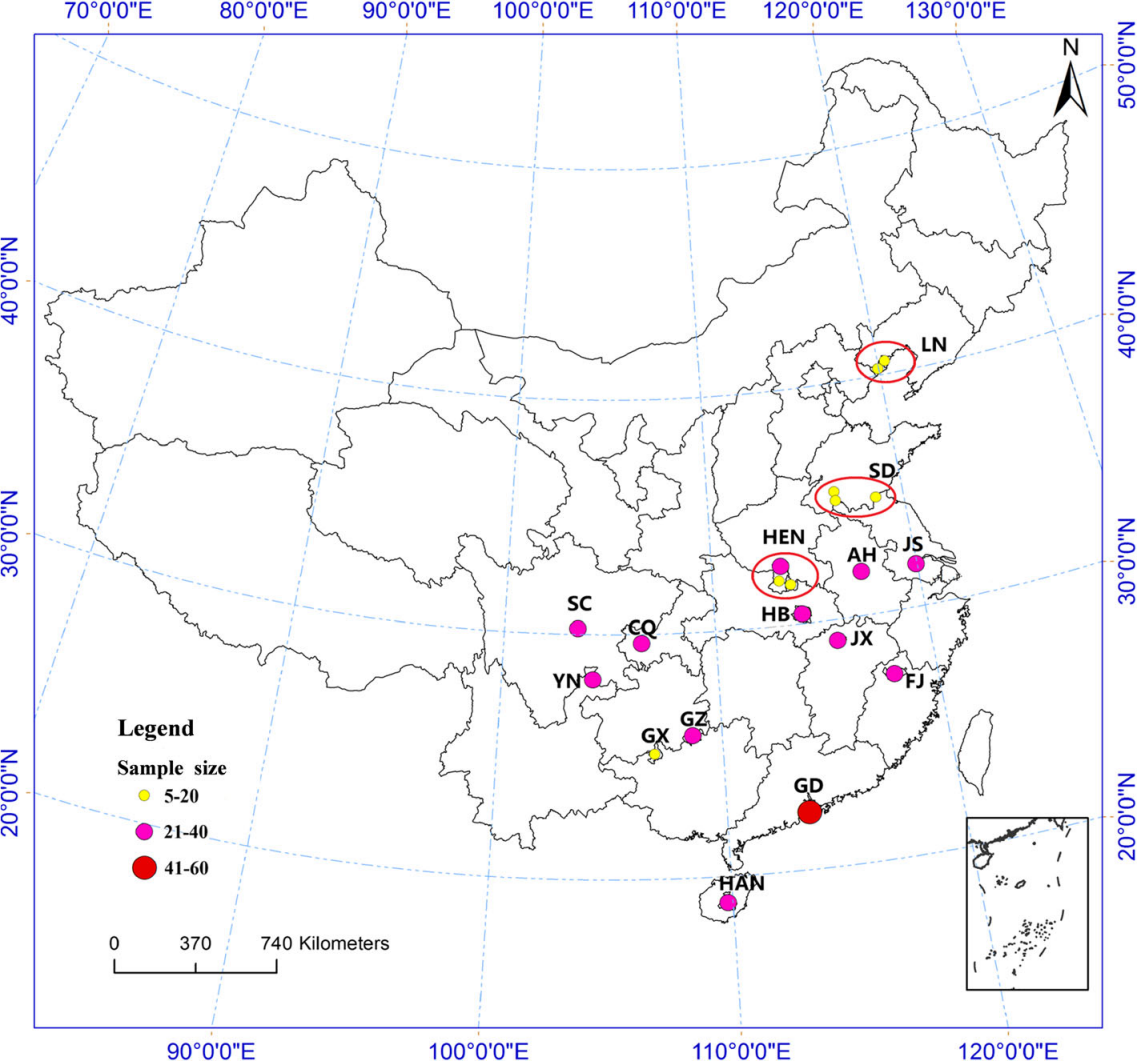
Schematic map of China showing sampling sites for *Anopheles sinensis*. The colour of the circles indicates the sample size of the collecting localities, the legend marked on the map (see Table 1 for abbreviations)

Wild mosquito adults were caught from July 1997 to August 2010 by light traps or artificial catching aspirator at livestock corrals. With the owners’ consent, the light traps were set up in pig or cow pens from 18:30 pm to 8:30 am. The number of sampling sites was 20 in 15 provinces from China. The collection information was summarized in Tables 1 and Fig. 1. Mosquitoes of the *An. hyrcanus* group were sorted out in the field by morphology using the identification keys [1], and kept individually in silica gel filled tubes at 4 °C until DNA extraction. The *An. sinensis* species identity was diagnosed by PCR assay based on ITS2 rDNA sequences [11].

**Table 1:** Collection information of *Anopheles sinensis* populations in this study

Population code	Collection site	Date	Coordinates	Sample size
AH	Hefei, Anhui	July 2006	31°49′N, 117°13′E	29
HB	Wuhan, Hubei	August 2006	30°35′N, 114°17′E	25
FJ	Jianyang, Fujian	September 1997	27°20′N, 118°06′E	30
CQ	Kaixian, Chongqing	July 2008	29°34′N, 106°32′E	24
HEN	Nanyang, Henan	August 2007	32°59′N, 112°31′E	39
	Guangshui, Hubei	June 2007	31°37′N, 113°49′E	6
	Shuizhou, Hubei	June 2007	31°41′N, 113°22′E	5
JS	Wujing, Jiangsu	July 1997	31°48′N, 119°58′E	40
GZ	Kaili, Guizhou	August 2007	26°34′N, 107°58′E	26
JX	Yongxiu, Jiangxi	September 2009	29°42′N, 109°83′E	28
GD	Zhuhai, Guangdong	October 2007	22°16′N, 113°34′E	46
SD	Jining, Shandong	July 2007	35°41′N, 116°34′E	14
	Yutai, Shandong	July 2000	35°01′N, 116°65′E	13
	Linshu, Shandong	July 2000	34°91′N, 118°66′E	10
HAN	Qiongzhong, Hainan	August 2010	39°28′N, 106°91′E	24
GX	Tiane, Guangxi	July 2005	24°99′N, 107°18′E	18
LN	Suizhong, Liaoning	August 2008	40°29′N, 120°01′E	7
	Xingcheng, Liaoning	August 2008	40°61′N, 120°75′E	8
SC	Pujiang, Sichuan	July 1997	30°19′N, 103°51′E	33
YN	Yanjin, Yunnan	July 2006	28°10′N, 104°23′E	28

### Genomic DNA isolation and sequencing of the *cox*1 mitochondrial gene fragments

The genomic DNA of single mosquitoes were isolated using QIAamp DNA Mini Kit (Qiagen, Hilden, Germany), following the manufacturer’s protocol. The forward (5′-GGT CAA CAA ATC ATA AAG ATA TTG G-3′) and reverse (5′-AAA CTT CAG GGT GAC CAA AAA ATC A-3′) primers were synthesized to amplify *cox*1 fragments [29]. The PCR kit was from Aidlab, China. PCR reactions were carried out in a Verity 96 well 157 Thermal Cycler (Applied Biosystems, Foster, USA). The cycling parameter included an initial step of denaturation at 94 °C for 5 min, followed by 30 cycles of amplification at 94 °C for 30 s, 45 °C for 30 s, and 72 °C for 30 s, with a final extension step at 72 °C for 5 min. After electrophoresis, PCR products were purified and sequenced in both directions using PCR primers on an ABI 3730 automatic sequencer (Applied Biosystems, Foster, USA) by Biosune Biotech (Shanghai Co., Ltd., Beijing, China). The electropherograms were inspected manually to verify sequence quality.

### Data analyses

Multiple *cox*1 sequence alignments were performed by CLUSTAL X [30] and edited by MEGA 6.0 [31]. The sequence differences within populations with number of haplotypes (H), the haplotype diversity (*Hd*), average number of nucleotide differences (*K*), average number of mutations per sequence (*θ*), number of variable sites (*S*) and nucleotide diversity (*Pi*) were estimated using DnaSP [32]. Median-joining networks of all *An. sinensis* haplotypes were constructed using Network 5.0 [33] to visualize relationships among unique haplotypes.

The genetic structure was analyzed with 15 populations. The percentage of sequence divergence within and between populations was calculated based on Nei & Li [34]. The pairwise *F*_ST_ values for short-term genetic distance between populations were estimated with the methods of Slatkin [35] and tested for significance by permutation. The gene flow [*N*m = (1 - *F*_ST_)/4 *F*_ST_]) between localities was estimated from pairwise *F*_ST_ [36]. Mismatch distributions and hierarchical analysis of molecular variance (AMOVA) were calculated using ARLEQUIN 3.11 [37]. Significant correlation between population genetic distance and linear straight geographical distances were assessed using the Mantel test implemented in Isolation by Distance Web Service (IBDWS) and significance was evaluated based on 1,000 permutations [38]. The neutrality test was evaluated by Tajima’s *D* and Fu’s *Fs*, which was estimated using the DnaSP software program [32].

## Results

### Population sampling and *An. sinensis* identification

Mosquito samples were collected from 20 sites in 15 provinces in China. Fifteen populations were analyzed, in which HEN, SD and LN consisted of specimens collected from two or three sites in proximity to each other (these were pooled, as stated in Table 1). There were 453 individuals of *An. sinensis* mosquitoes, which were identified by PCR assay.

### Sequence characteristics of *cox*1 gene

A segment of mtDNA, corresponding to the coding region of *cox*1, was successfully amplified from *An. sinensis* individuals. A sequence of 662 nt was obtained and analyzed. No insertion or deletion was detected across all samples. The conserved sites were 553, variable sites 107. Of these, 41 were singleton and 66 were parsimony-informative.

The average number of nucleotide differences (*K*) ranged from 2.61 (LN) to 6.97 (HAN), corresponding with the range of nucleotide diversity (*Pi*) and average number of mutations (*θ*), which were 0.00394 ± 0.00084 (mean ± standard deviation, SD) (LN) - 0.01054 ± 0.00145 (HAN) and 0.007 (LN/GX) - 0.019 (HB), respectively (Table 2).

**Table 2:** Summary data for populations, haplotypes and nucleotide diversity of *Anopheles sinensis*

Population code^a^	Sample size	H/Percentage	*S*	*K*	*θ*	*Hd* ± SD	*Pi* ± SD
AH	29	28/96.55	37	5.26	0.015	0.998 ± 0.010	0.00795 ± 0.00105
CQ	24	21/87.50	26	4.68	0.011	0.989 ± 0.015	0.00708 ± 0.00113
FJ	30	26/86.67	32	5.51	0.013	0.989 ± 0.013	0.00833 ± 0.00114
GD	46	31/67.39	35	5.11	0.012	0.974 ± 0.011	0.00772 ± 0.00093
GX	18	12/66.67	16	3.66	0.007	0.935 ± 0.041	0.00553 ± 0.00105
GZ	26	23/88.46	31	5.37	0.014	0.982 ± 0.020	0.00811 ± 0.00128
HAN	24	19/79.17	28	6.97	0.012	0.960 ± 0.031	0.01054 ± 0.00145
HEN	50	42/84.00	53	6.71	0.012	0.993 ± 0.005	0.01014 ± 0.00099
HB	25	22/88.00	29	4.84	0.019	0.987 ± 0.017	0.00731 ± 0.00118
JS	40	33/82.50	41	4.67	0.015	0.983 ± 0.012	0.00706 ± 0.00081
JX	28	24/85.17	28	4.68	0.011	0.981 ± 0.018	0.00707 ± 0.00068
LN	15	10/66.67	14	2.61	0.007	0.914 ± 0.056	0.00394 ± 0.00084
SD	37	28/75.68	35	6.28	0.010	0.973 ± 0.016	0.00949 ± 0.00122
SC	33	17/51.52	26	3.42	0.013	0.911 ± 0.032	0.00516 ± 0.00066
YN	28	10/35.71	25	6.01	0.010	0.709 ± 0.091	0.00908 ± 0.00207

*Abbreviations*: *H* number of haplotypes, *S* the number of segregating sites, *K* the average number of nucleotide differences, *θ* the average number of mutations per sequence, *Hd* the haplotypes diversity, *Pi* is nucleotide diversity

^a^Population codes as in Table 1

**Fig. 2:**
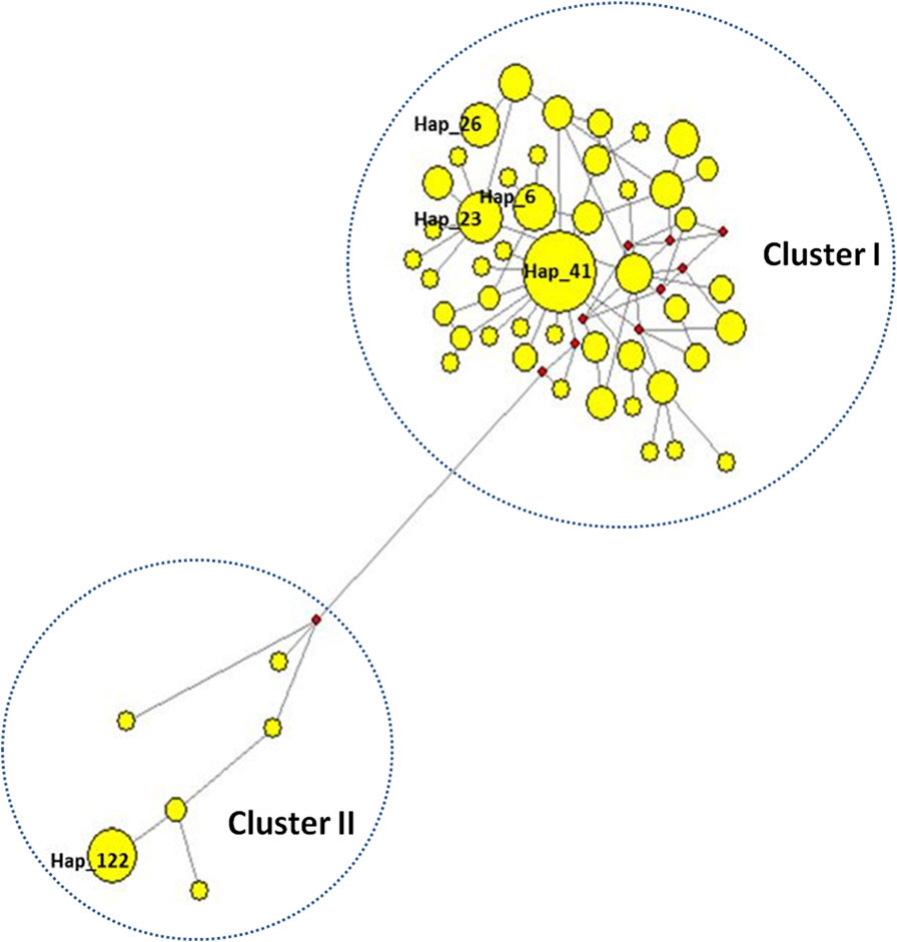
Haplotype network of the *cox*1 as calculated by Network 5.0. Each circle (*yellow*) represents a haplotype, and the size of a circle is proportional to the number of individuals that contained the haplotype, the *red* dots (median vector) are a hypothesised haplotype, which was not detected

A total of 247 haplotypes (GenBank accession numbers: KX779529–KX779775) were detected in 453 (247/453; 54.53%) of the *An. sinensis* individuals (Additional file 1: Table S1). The range of haplotypes diversity (*Hd*) was from 0.709 ± 0.091 (mean ± standard deviation, SD) (YN) to 0.998 ± 0.010 (AH), corresponding to the percentage of the haplotype from 35.71% (YN) to 96.55% (AH). There were five abundant haplotypes, containing more than 10 individuals, as Hap_41 (*n* = 37), Hap_122 (*n* = 16), Hap_23 (*n* = 14), Hap_6 (*n* = 13) and Hap_26 (*n* = 10). Forty-one haplotypes occurred in more than one population, the frequency of which was 16.6% (41/247). Few haplotypes were not detected. The genealogic network showed that the haplotypes were divided into two clusters (Fig. 2). Cluster I included 241 detected haplotypes at a high level of homoplasy, Hap_41 being the central haplotype. Cluster II included seven haplotypes, Hap_122 being considered central. The samples in haplotype Hap_122 were almost all from the YN population, except one from HEN. The individuals of the other five detected haplotypes were from AH, HEN, HB, JS and GZ populations.

### Genetic variation among populations

The fixation index (*F*_ST_) was used to evaluate the genetic distance among populations. In this study there were seven negative *F*_ST_ values, comprising AH/FJ, CQ/JX, FJ/GZ, FJ/HEN, FJ/JS, HB/GZ and HB/JS, which indicated genetic differentiation among these populations was very limited (Table 3). Across all populations, *F*_ST_ values of the YN population were all greater than 0.45, and correspondingly, the gene flow (*N*m) of the YN population were all much less than 1.0. In theory, it was hard to prevent genetic divergence caused by genetic drift if the gene flow (*N*m) value was less than 1.0 [36].

**Table 3:** Pairwise genetic distance (*F*_ST_) and gene flow (*N*m) for populations of *Anopheles sinensis* in China*.* Below diagonal were genetic distance (*F*_ST_) and above were gene flow (*N*m) among populations

Population code^a^	AH	CQ	FJ	GD	GX	GZ	HAN	HB	HEN	JS	JX	LN	SC	SD	YN
AH		6.509	-28.152	3.979	7.570	18.407	10.325	5.039	346.972	11.857	5.258	2.569	1.261	5.725	0.222
CQ	0.037		11.319	4.078	10.605	10.167	2.716	8.547	8.440	17.866	-30.812	10.931	1.344	11.454	0.201
FJ	-0.009	0.022		13.608	13.842	-15.193	5.359	41.909	-43.503	-117.072	10.276	3.974	1.434	15.239	0.232
GD	0.059	0.058	0.018		4.037	25.028	2.187	44.795	6.385	11.145	4.773	2.951	1.171	4.663	0.223
GX	0.032	0.023	0.018	0.058		26.233	3.832	13.570	10.038	88.089	11.346	10.797	1.294	5.642	0.187
GZ	0.013	0.024	-0.017	0.010	0.009		5.022	-37.844	51.942	89.036	13.426	5.108	1.597	11.356	0.228
HAN	0.024	0.084	0.045	0.103	0.061	0.047		2.512	7.064	2.753	2.711	1.392	1.400	3.339	0.285
HB	0.047	0.028	0.006	0.006	0.018	-0.007	0.091		24.164	-40.638	7.768	7.003	1.560	9.581	0.229
HEN	0.001	0.029	-0.006	0.038	0.024	0.005	0.034	0.010		26.459	6.385	3.594	1.617	12.730	0.298
JS	0.021	0.014	-0.002	0.022	0.003	0.003	0.083	-0.006	0.009		9.839	11.471	1.317	9.880	0.213
JX	0.045	-0.008	0.024	0.050	0.022	0.018	0.084	0.031	0.038	0.025		9.516	1.343	6.442	0.201
LN	0.089	0.022	0.059	0.078	0.023	0.047	0.152	0.034	0.065	0.021	0.026		1.163	3.132	0.153
SC	0.165	0.157	0.148	0.176	0.162	0.135	0.152	0.138	0.134	0.160	0.157	0.177		0.947	0.156
SD	0.042	0.021	0.016	0.051	0.042	0.022	0.070	0.025	0.019	0.025	0.037	0.074	0.209		0.284
YN	0.529	0.554	0.519	0.529	0.572	0.523	0.467	0.522	0.456	0.540	0.555	0.620	0.616	0.469	

^a^Population codes as in Table 1

In the hierarchical AMOVA, both the ‘among populations’ and ‘within populations’ variance components were considerably high, the latter (83.83%) contributing more to total variances than the former (Table 4). The mean genetic divergence among populations was 0.16 by *cox*1 sequences.

**Table 4:** AMOVA analysis of genetic variation in *Anopheles sinensis* populations by mitochondrial *cox*1 gene. Mean *F*_ST_ = 0.16

Source of variation	Degrees of freedom	Variance components	Variation (%)
Among populations	14	0.50	16.17
Within populations	438	2.61	83.83
Total	452	3.12	100

**Fig. 3:**
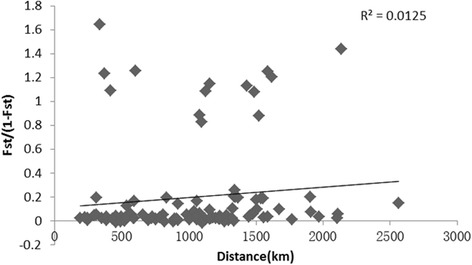
Correlation of the genetic variation and geographical distance for pairwise comparisons of *Anopheles sinensis* populations

Tests of isolation by distance were performed for all of the populations. No statistically significant correlations were detected between genetic differentiation and geographical distances based on the Mantel test (Fig. 3). The correlation coefficient was 0.11, which is not significant based on 1,000 permutations (*P* = 0.59). The results suggested that geographical distance does not significantly contribute to the genetic differentiation observed in *An. sinensis* populations.

### Neutrality test and mismatch analysis

The Tajima’s *D* and Fu’s *Fs* values were all negative (Table 5) by DnaSP software, which suggested many low-frequency mutations in populations as well as that the populations were in the process of expansion. The strongly negative values of Fu’s *Fs* were observed (*P* < 0.01) in each population, which would be more sensitive in detecting deviations from neutrality.

**Table 5:** Mismatch and neutrality tests results of *Anopheles sinensis* populations from China

Population code	Neutrality tests		Mismatch analysis	
	Tajima’s *D*	Fu’s *F*_*S*_	SSD	Rag
AH	-1.63	-25.48	0.01	0.03
CQ	-1.22	-25.46*	0	0.03
FJ	-1.15	-25.41*	0.01	0.02
GD	-1.22	-25.51*	0.01	0.02
GX	-0.81	-19.6*	0.07	0.08
GZ	-1.26	-25.43*	0.07	0.02
HAN	-0.26	-21.4*	0	0.01
HEN	-1.5	-25.12*	0.07*	0.01
HB	-1.38	-25.57*	0.08	0.01
JS	-1.81	-25.63*	0.03	0.02
JX	-1.27	-25.65*	0	0.02
LN	-1.56	-17.64*	0.07	0.04
SD	-0.88	-25.22*	0.01	0.01
SC	-1.64	-26.09*	0.02	0.04
YN	-0.23	-25.3*	0.04	0.06
Mean	-1.19	-24.3*	0.03	0.03
SD	0.45	2.48	0.03	0.02

*Abbreviations*: *SSD* sum of squared deviation, *Rag* raggedness index

*P < 0.01

Demographic expansions were analyzed using mismatch analysis; both the sum of squared deviation (SSD) values (0.03, *P* = 0.24) and raggedness index (Rag) (0.03) were not statistically significant in almost all the populations except the SSD value for the HEN population (Table 5) [39]. The mismatch distributions showed a smooth and main unimodal curve peaks (Additional file 2: Figure S1), which coincide with the population expansion model.

## Discussion

In this study, the population genetic diversity was analyzed on *An. sinensis* samples obtained from 20 collection sites (22°16′N to 40°61′N, 103°51′E to 120°75′E), which covered almost the entire distribution range of *An. sinensis* in China [1]*.* Sample sizes and site distribution throughout China were adequate and provided an ample dataset to study. The results should be an objective representation, since sampling strategy and geographical coverage greatly influence the analysis and interpretation of the data generated from the samples.

There is considerable *cox*1 gene nucleotide diversity in anopheline mosquitoes, which has been used to explore population genetic structure [21–26] and DNA barcoding [27, 40–44]. The results showed that genetic polymorphism of *An. sinensis* populations have a high haplotype diversity (*Hd* = 0.709–0.998) and nucleotide diversity (*Pi* = 0.004–0.011), in turn suggesting that *cox*1 can be considered a suitable molecular marker for calculating genetic variation and detecting genetic structure. The high level of genetic diversity indicated that the species could maintain a relatively large effective population size by a broad tolerance to environmental and habitat pressure. Due to the evolutionary rates of different molecular markers, the genetic divergence showed different degrees, e.g. *Pi* values as 0.61−1.00 of *cox*1 gene, compared to 0.24−0.65 of *cox*2 in *An. sinensis* samples [27]; *θs* values as 0.58−4.285 of *cox*2, while as 0.274−3.545 of *cytb* in *An. lesteri* populations [45].

Overall, the results showed that there were low genetic differences and high gene flow among different populations except in the case of the YN population, which was similar with that previously detected in *An. sinensis* populations in China using microsatellites and other molecular markers. There was also no correlation between genetic differences and geographical distance [16, 18, 19, 46]. The distribution range of *An. sinensis* in China was wide, with a large population size and similar ecological habit [1]. Gene flow and introgression between individuals occurred easily, the expansion and spread of genes responsible for immunity against malaria or insecticide resistance thus highly probable between the populations [20, 47]. Yunnan, a mountainous area in southwest China, is noted as a center of biodiversity because of its highly complex topography [48, 49]. It was obvious that the YN population of *An. sinensis* was relatively unique in this study as in other reports [16, 46]. The finding also reported that there was a great difference between YN and other populations in the pyrethroid resistance mechanism [47, 50]. Therefore, both physical distance and heterogeneous landscape could be factors inhibiting gene flow between the YN population and others. In the Republic of Korea (ROK), *An. sinensis* populations represented positive and significant isolation-by-distance patterns by microsatellites and mtDNA control region, and both molecular markers showed the Taebaek and Sobaek Mountain ranges to be barriers between the northern and southern parts of the ROK [17, 51].

The genealogy network showed that these haplotypes of *An. sinensis* were divided into two clusters. Cluster I included all samples from 15 populations and cluster II included most samples from the YN population, this supported by the phylogenetic findings showing the YN populations also as an independent clade (Additional file 3: Figure S2). However, three clusters of *An. sinensis* populations were detected over seven Chinese provinces by mtDNA-*ND5* gene. All three clusters were observed in *An. sinensis* samples collected from different sites demonstrating apparent differences in relative abundance for given clusters [16]. The CI was similar with cluster I, while CII and CIII were added together corresponding to cluster II of this study. But two gene pools grouping *An. sinensis* samples by microsatellites were difficult to correspond to the two clusters by *cox*1 in the present study [19].

*Anopheles sinenesis* population genetic variation detected by the microsatellites and the mtDNA were compared. Firstly, microsatellite results sorted the Chinese *An. sinensis* populations into two gene pools, six of 14 populations were mixed with individuals from both gene pools, indicating the coexistence of two genetic units in the areas sampled [19]. In this study, 15 populations were divided into two clusters, and almost all individuals in cluster II were from the YN population. Secondly, the level of *F*_ST_ values in *An. sinensis* populations from China used by microsatellites showing a range of 0.004−0.048 [19] compared to a range of 0.000−0.452 in this study. Similar the report in ROK, the range of *F*_ST_ was 0.000−0.110 by microsatellites [51], while 0.000−0.3125 by mtDNA control region [17]. Thirdly, no overall correlation between genetic and geographical distance was detected in populations of *An. sinensis* in China, both by mtDNA and microsatellites, unlike in ROK [17, 51]. This may be due to China’s wide geographical area, resulting in dilution of the impact imposed by geographical barriers.

In this study, the neutrality test values of Tajima’s *D* and Fu’s *Fs* were all negative, which suggensted that *An. sinensis* population expansion events may have occurred in the demographic history*.* Moreover, the distribution of pairwise nucleotide differences was characteristic of a population that has undergone a large expansion. Furthermore, small and not statistically significant mismatch analysis statistics SSD and Rag supported the hypothesis of population expansion. These findings were also consistent with previously reported results based on the *ND5* gene [16]. These results were supported by many low-frequency mutations in populations and with possible effects of purifying selection, or population expansion of *An. sinensis* in these locations.

## Conclusions

A better understanding of genetic diversity of local *An. sinensis* and metapopulation dynamics could provide important information for the epidemiological surveillance and malaria vector control strategy. In this study, our collection sites covered most of the distribution in which malaria used to be meso-endemic or hypo-endemic in its regions in China. *Anopheles sinensis* populations showed high genetic polymorphism by *cox*1 gene. The weak genetic structure may be a consequence of low genetic differentiation and high gene flow among populations, except in the case of the YN population. The population structure implies that the expansion and spread of genes responsible for immunity against malaria or insecticide resistance would be more probable between the populations. The YN population was isolated from the other populations with its large pairwise *F*_ST_ value, gene flow limited by geographical distances and barriers, thus forming a separate cluster (cluster II). *Anopheles sinensis* population size was large owing to the recent expansion of these populations in China.

## Additional files

Additional file 1: Table S1. GenBank accession numbers for the *Anopheles sinensis* haplotypes among populations. (PDF 2557 kb)

Additional file 2: Figure S1. Graphs of the mismatch distributions analysis for total populations of *Anopheles sinensis* using DnaSP 5.10. The X axis shows the observed distribution of pairwise nucleotide differences and the Y axis shows the frequencies. The dotted lines represent the observed frequency of pairwise differences, and the solid lines show the expected values under the sudden population expansion model. (TIF 8 kb)

Additional file 3: Figure S2. Phylogenetic tree of *Anopheles sinensis* populations based on mitochondrial *cox*1 gene. (TIF 512 kb)

## Abbreviations

*cox*1: Cytochrome *c* oxidase subunit I
*cox*2: Cytochrome *c* oxidase subunit II
*F*_ST_: Fixation index
ITS2: Internal transcribed spacer 2
*nad*5: NADH dehydrogenase subunit 5
*N*m: Gene flow
PCR: Polymerase chain reaction
RAPDs: Random amplified polymorphic DNA
SD: Standard deviation
