# Erratum to: Genetic diversity and population structure of the primary malaria vector *Anopheles sinensis* (Diptera: Culicidae) in China inferred by *cox*1 gene

**DOI:** 10.1186/s13071-017-2048-1

**Published:** 2017-03-02

**Authors:** Xinyu Feng, Libin Huang, Lin Lin, Manni Yang, Yajun Ma

**Affiliations:** 1 WHO Collaborating Center for Tropical Diseases, Key Laboratory of Parasite and Vector Biology, Ministry of Public Health, National Institute of Parasitic Diseases, Chinese Center for Disease Control and Prevention, Shanghai, 200025 China; 2 Department of Tropical Infectious Disease, Second Military Medical University, Shanghai, 200433 China; 3 Second Military Medical University Press, Shanghai, 200433 China

## Erratum

After the publication of this work [1] it was noticed that there were errors in a number of coordinates in Table 1. The corrected table is shown below with the updated coordinates for population codes JX, SD, HAN, GX and LN.

**Table 1:** Collection information of *Anopheles sinensis* populations in this study

Population code	Collection site	Date	Coordinates	Sample size
AH	Hefei, Anhui	July 2006	31°49′N, 117°13′E	29
HB	Wuhan, Hubei	August 2006	30°35′N, 114°17′E	25
FJ	Jianyang, Fujian	September 1997	27°20′N, 118°06′E	30
CQ	Kaixian, Chongqing	July 2008	29°34′N, 106°32′E	24
HEN	Nanyang, Henan	August 2007	32°59′N, 112°31′E	39
	Guangshui, Hubei	June 2007	31°37′N, 113°49′E	6
	Shuizhou, Hubei	June 2007	31°41′N, 113°22′E	5
JS	Wujing, Jiangsu	July 1997	31°48′N, 119°58′E	40
GZ	Kaili, Guizhou	August 2007	26°34′N, 107°58′E	26
JX	Yongxiu, Jiangxi	September 2009	29°01′N, 109°48′E	28
GD	Zhuhai, Guangdong	October 2007	22°16′N, 113°34′E	46
SD	Jining, Shandong	July 2007	35°41′N, 116°34′E	14
	Yutai, Shandong	July 2000	35°01′N, 116°38′E	13
	Linshu, Shandong	July 2000	34°55′N, 118°38′E	10
HAN	Qiongzhong, Hainan	August 2010	19°28′N, 106°51′E	24
GX	Tiane, Guangxi	July 2005	25°06′N, 107°10′E	18
LN	Suizhong, Liaoning	August 2008	40°29′N, 120°01′E	7
	Xingcheng, Liaoning	August 2008	40°36′N, 120°45′E	8
SC	Pujiang, Sichuan	July 1997	30°19′N, 103°51′E	33
YN	Yanjin, Yunnan	July 2006	28°10′N, 104°23′E	28
