# Supplementary material for: Genetic diversity and population structure of the primary malaria vector Anopheles sinensis (Diptera: Culicidae) in China inferred by cox1 gene
Source: Parasit Vectors. 2017 Feb 10;10:75. doi: 10.1186/s13071-017-2013-z (PMC5439230; doi:10.1186/s13071-017-2013-z)
Supplement: Supplementary file 1 — GenBank accession numbers for the Anopheles sinensis haplotypes among populations. (PDF 2557 kb) [file 13071_2017_2013_MOESM1_ESM.pdf]

Additional file 1: Table S1. Polymorphic positions of COI (mtDNA) of An. sinensis collected from China

H		N	AN
	12236677888911111111111122222222222222222233333333333333333333444444444444444444555555555555555556666666666		
	74652514059201556677888900002345556778889900112334445556677999001125556677788899012233334455689990000122333		
	47254703368103697201799230140423149281470692917278170982584746915617435836792817610362568403258		
Hap_1	CGATATTTTGTTTGATTAACTGTAAACTGAAATCAACCTAAGAGAGCAATATTTAAGTTTCTGAAATAATATGTATTCTAGGCTTACAACATTTCACCAGTATTTAT	5	KX779529
Hap_2	....G......................................	1	KX779530
Hap_3	............................A...A....................G.....	1	KX779531
Hap_4	............................A.............G..	2	KX779532
Hap_5	............................A...G................	4	KX779533
Hap_6	............................A................A...T.........	13	KX779534
Hap_7	............................A.................T..	1	KX779535
Hap_8	............................A.................T..T.................	6	KX779536
Hap_9	............................A......G..........T..C...	1	KX779537
Hap_10	............................A......G..........T..	4	KX779538
Hap_11	............................A......G..........T..T....C....	1	KX779539
Hap_12	............................A......G..........T...C....	4	KX779540
Hap_13	............................A.................T...............................C.....................C.C....	1	KX779541
Hap_14	............................A................AT..T..........C.C....	3	KX779542
Hap_15	............................A.................T....................G..................................C....	6	KX779543
Hap_16	............................A.................T...C....	9	KX779544
Hap_17	..............G.............A.................T..........................C...T........................C....	6	KX779545
Hap_18	..............G............CA................AT..............................T........................C....	1	KX779546
Hap_19	............................A.................T..............................T...................T....C....	1	KX779547
Hap_20	..............G.............A.................T.............................C.........................C.C..	1	KX779548
Hap_21	..............G.............A.................T...C.C..	1	KX779549
Hap_22	..............G.............A.................T..C......C....	1	KX779550
Hap_23	..............G.............A.................T...C....	14	KX779551
Hap_24	..............G.............A............A....T....................................C..................C....	1	KX779552
Hap_25	..............G.............A.................T...G...........C....	2	KX779553
Hap_26	......C.......G...........T.A...C....	10	KX779554
Hap_27	...........C..G....T......T.A..................G......G..............CG..........................T.........	2	KX779555
Hap_28	..............G....T.....GT.A.................TG......G.......A..G....G.......................T.......CG...	1	KX779556
Hap_29	..............G...........T.A...C....	1	KX779557
Hap_30	......C.......G...........T.A...............C.................A.............CT........................C....	1	KX779558
Hap_31	..............G....T........A.................T...C....	2	KX779559
Hap_32	.......C......G.............A.................T.....................G....C...T........................C....	1	KX779560
Hap_33	....T..................G...	2	KX779561
Hap_34	....T..	6	KX779562
Hap_35	..........................T.A......G.....A....T.........A...C....	1	KX779563
Hap_36	......A.....................A......G..........T..	1	KX779564
Hap_37	............................A.................T...T.......C....	1	KX779565
Hap_38	............................A.................T....................G................................C.C....	5	KX779566
Hap_39	............................A................AT...C....	1	KX779567
Hap_40	............................A...............G.T.................G.....................................C....	1	KX779568
Hap_41	............................A..	37	KX779569
Hap_42	............................A................................C...........................T.................	1	KX779570
Hap_43	............................A.................................A.G..	1	KX779571
Hap_44	............................A................A................A..................................T.........	1	KX779572
Hap_45	............................A................A...	6	KX779573
Hap_46	............................A................A..T............	1	KX779574
Hap_47	............................A..T...................T.........	3	KX779575
Hap_48	..............G.............A..T....C....	1	KX779576
Hap_49	.......C...C..G....T......T.A.................TG......G.......A......CG....................................	1	KX779577
Hap_50	...........C..G....T......T.A..................G......G..............CG..................T....T........A...	1	KX779578
Hap_51	.....C.....C..G....T......T.A..................G......G..............CG................................A...	2	KX779579
Hap_52	.....C.....C..G....T......T.A..................G......G..............CG.....C..........................A...	1	KX779580
Hap_53	.....C.....C..G....T......T.A..................G......G.......A......CG..........................T.....A...	1	KX779581
Hap_54	..............G.............A.................T..........................C...T.................C.T....C....	1	KX779582
Hap_55	............................A.................T..............................T........................C....	1	KX779583
Hap_56	............................A.................TG....C.........A.......................................C....	1	KX779584
Hap_57	............................A...C.............TG....C...C....	1	KX779585
Hap_58	................C...........A...C.............T...C....	1	KX779586
Hap_59	............................A.................T..GC....	4	KX779587
Hap_60	............................A.............G..A...T.........	2	KX779588
Hap_61	..........C.................A................A...	1	KX779589
Hap_62	................C...........A................A...	1	KX779590
Hap_63	............................A...C...G................	1	KX779591
Hap_64	............................A...C..T.........	1	KX779592
Hap_65	............................A.....................G.C..	1	KX779593
Hap_66	.....C......................A.......................C..T.........	1	KX779594
Hap_67	............................A..T.............................	3	KX779595
Hap_68	....T...C......	1	KX779596
Hap_69	....T.....................................A......................	1	KX779597
Hap_70	..........................T...................T..C.............	2	KX779598
Hap_71	....T..T.........	2	KX779599
Hap_72	....T...	2	KX779600
Hap_73	....AG..	1	KX779601
Hap_74	............................A......GT...C....	1	KX779602
Hap_75	............................A................A........G...C....	3	KX779603
Hap_76	..............G.............A...C....	8	KX779604
Hap_77	..............G.............A.................T..................G....................................C....	1	KX779605
Hap_78	..............G.............A...C..............................	1	KX779606
Hap_79	............................A...C....	6	KX779607
Hap_80	............................A..................C.....................C.....................................	2	KX779608
Hap_81	............................A.........................G..	1	KX779609
Hap_82	..........C.................A..T.............................	2	KX779610
Hap_83	............................A...C.........G..AT..T............C....	1	KX779611
Hap_84	............................A...C............AT.......G................A.....T...........T..........C.C....	1	KX779612
Hap_85	............................A................................C...G...	1	KX779613
Hap_86	............................A................................C..C..	1	KX779614
Hap_87	............................A.................................A..	1	KX779615
Hap_88	.......G....................A..C...........	1	KX779616
Hap_89	............................A................A........G..T.........	2	KX779617
Hap_90	............................A...C............A...	1	KX779618
Hap_91	............................A.................T....................G.......................................	1	KX779619
Hap_92	............................A................AT..T..........C......	1	KX779620
Hap_93	............................A.................T...............T..........................T.....G....C.C....	1	KX779621
Hap_94	...........C..G...........T.A.................T....C..C....	1	KX779622

Hap_95	.............G..........T.A...........A...T...C....	1	KX779623
Hap_96	.............G...........CA...............T............................T................G.T....C....	1	KX779624
Hap_97	.....C.C...C..G....T.....T.A...............TG............A.....CG..............................A...	1	KX779625
Hap_98	.............G....T.......A...............AT................G...CG.....T...............T.......CG...	1	KX779626
Hap_99	T....C.....C..G....T.....T.A.................G.....G.............CG..............................A...	1	KX779627
Hap_100	.....C.....C..G....T.....T.A............G....G.....G.............CG..............................A...	1	KX779628
Hap_101	.............G............A...........A...AT........A..C....	1	KX779629
Hap_102	.............G............A...C...........T..G...C....	2	KX779630
Hap_103	.............G............A...C...........TG...G...C....	1	KX779631
Hap_104	.............G...........CA...............TG...........................T.......................C....	1	KX779632
Hap_105	..........................A...........A...AT........A............................T.........C.C....	1	KX779633
Hap_106	..........................A...C...........AT.............A.......................T.........C.C...C	1	KX779634
Hap_107	..........................A...C...........AT......................................T.........C.C....	1	KX779635
Hap_108	..........................A...C...........AT......................................T.........C....	1	KX779636
Hap_109	..........................A.......T.......T..............A.........................T.........C.C....	1	KX779637
Hap_110	..........................A...............AT...........................T.......................C....	1	KX779638
Hap_111	..........................A............G....C..	1	KX779639
Hap_112	..........................A.................................C....................................	1	KX779640
Hap_113	.........................T.A...T........T.............CA...	1	KX779641
Hap_114	..........C...G...........A...............T........................C...T.......................C....	1	KX779642
Hap_115	.............G............A...............T..T....C....	2	KX779643
Hap_116	.............G............A..........................C...C....	1	KX779644
Hap_117	......C.......GA.........T.A...C..C....	1	KX779645
Hap_118	......C.......GA.........T.A..C....	1	KX779646
Hap_119	......C.......G....T.....T.A...C..........................C....	1	KX779647
Hap_120	..........C..G....T.....T.A.................G.....G.............CG.....................T.....A...	1	KX779648
Hap_121	..........C..G....T.....T.A................TG.....G.............C..CG..................T............A..C	1	KX779649
Hap_122	.....C.....C..G....T.....T.A...........G....T.....G...........G..G..................G...........A...	16	KX779650
Hap_123	..........C......GT......T.A...................G....................CG..............................A...	1	KX779651
Hap_124	.............G....T......T.A.....................G.............G....G......................T..T....CA...	1	KX779652
Hap_125	.............G....T......T.A.................G.................G....G......................T.......C....	1	KX779653
Hap_126	.............G....T.....GT.A...............A.................G..........T...............T.......CG...	1	KX779654
Hap_127	..........................A...G.	1	KX779655
Hap_128	..........................A...C..........	1	KX779656
Hap_129	..........................A............G..........................G...........................	1	KX779657
Hap_130	..........................A.......................................G......................T........	1	KX779658
Hap_131	..........................A..........G.....T..................G.................................C....	1	KX779659
Hap_132	..........................A...............T......C.............G.................................C....	1	KX779660
Hap_133	..........................A...........A...AT...............G...G...............................C.C....	1	KX779661
Hap_134	..........................A...............T..........C...........................T...........C....	1	KX779662
Hap_135	..........................A...............T..............T.........................T.........C.C....	1	KX779663
Hap_136	.......C.............G....A..	1	KX779664
Hap_137	.............G....T....G.T.A.................G.....G......A..G....................T.......T....C.......	1	KX779665
Hap_138	..........................A.....G.........T...........................T.......................C....	1	KX779666
Hap_139	........C.................A............G..A..............................A.............T.........	1	KX779667
Hap_140	......................C.....A..	2	KX779668
Hap_141	..........................A...G................	2	KX779669
Hap_142	..........................A...T.........	3	KX779670
Hap_143	......................C.....A...............A.G...	1	KX779671
Hap_144	..........................A...............A.....................G...................................	1	KX779672
Hap_145	..........................A.......................................G..................................	1	KX779673
Hap_146	..........................A...............T.........................C............................C....	1	KX779674
Hap_147	.............G............A...C...........T..C....	1	KX779675
Hap_148	.............G............A...............T.........................T...................G.......C....	1	KX779676
Hap_149	......C.......G..........T.A...G.......C....	1	KX779677
Hap_150	..G....C.......G..........T.A......................G...C....	1	KX779678
Hap_151	..........C..G....T.....T.A.................G.....G.............CG................................	1	KX779679
Hap_152	..................T......T.A.................G..G.............G....G.............G........T..T....CA...	1	KX779680
Hap_153	......C.......G..........T.A...C..........................C....	1	KX779681
Hap_154	......C.......G..........T.A...............A..C....	1	KX779682
Hap_155	..........................A...............................A...........................G............	1	KX779683
Hap_156	.C...............G.........A.G...G........................A......................................	1	KX779684
Hap_157	.........A................A..	1	KX779685
Hap_158	..........................A..G...	1	KX779686
Hap_159	..........................A...C........G...	1	KX779687
Hap_160	....T............	1	KX779688
Hap_161	..............................A..T.......................	1	KX779689
Hap_162	..........................A...............A................C.....................................	1	KX779690
Hap_163	..G.......................A...............A..	1	KX779691
Hap_164	..........................A...............A...............................A..............T.........	1	KX779692
Hap_165	............A.............A...T.........	1	KX779693
Hap_166	.............G............A...............T....................C......C..........................C....	1	KX779694
Hap_167	.............G............A...............T..GC.C..	1	KX779695
Hap_168	.............G............A...C...T.......................C....	1	KX779696
Hap_169	......................G....A...............T..C....	1	KX779697
Hap_170	..G.......................A...............T...T....C....	1	KX779698
Hap_171	.....C.....C..G....T.....T.A.................G.....G.............CG................................	1	KX779699
Hap_172	.............G....T.....GT.A...............TG..G...............G..................T..T...........	1	KX779700
Hap_173	..........................A.G.........A...T..................G...................................C....	1	KX779701
Hap_174	.............G............A...C...........T.........................C............................C....	1	KX779702
Hap_175	.............G............A...C...........T.......C.........G.....................................C....	1	KX779703
Hap_176	..........................A...C.......A...T............................T.......................C....	1	KX779704
Hap_177	..........................A...........................C..	2	KX779705
Hap_178	..........................A...C..	1	KX779706
Hap_179	..........................A.............................CA........................T...............	1	KX779707
Hap_180	..........................A.................C.....G...............................G................	1	KX779708
Hap_181	......................C.....A...........A..	1	KX779709
Hap_182	......................................A......................G...................................	1	KX779710
Hap_183	..........................A.G.........A...A...	1	KX779711
Hap_184	..........................A...........A...A.......G..	1	KX779712
Hap_185	..........................A............G..AT...C....	1	KX779713
Hap_186	.................G........A...............T..C....	1	KX779714
Hap_187	..........................AG..........A..T.T..................C..................................C....	1	KX779715
Hap_188	.............G..........T.A...........A...T...C............C....	1	KX779716
Hap_189	.............G..........T.A.G......G..A..T...C....	1	KX779717
Hap_190	..........................A.....................................A................................C....	4	KX779718
Hap_191	..........................A...........A..C....	1	KX779719
Hap_192	..........................A...G....	1	KX779720
Hap_193	.............G...........GA...........G..	1	KX779721
Hap_194	...C.................G....A...............T..C....	1	KX779722
Hap_195	..........................A...............AT..T....C.C..	1	KX779723
Hap_196	....G........G.....C.....CA...............T.........A...............T.................T....C....	1	KX779724
Hap_197	....G.......C.G.....C.....CA...............T.........A...............T.................T....C....	1	KX779725
Hap_198	....G........G.....C.....CA...............T.......G.................CT..............................C....	1	KX779726
Hap_199	....................T......A...	1	KX779727

Hap_200	...........................A...............................C................G..........................	1	KX779728
Hap_201	...........................A...C.............................	1	KX779729
Hap_202	..................T........A...	1	KX779730
Hap_203	...........................A...........A...	5	KX779731
Hap_204	..........................GA...........A...	2	KX779732
Hap_205	...........................A...........A....................A...........A..............................	1	KX779733
Hap_206	..............G............A...........A...	1	KX779734
Hap_207	...........................A........T..T.........	1	KX779735
Hap_208	...........................A........T.......T...................G......................................	1	KX779736
Hap_209	...........................A...C...T........T...............A..	1	KX779737
Hap_210	..............G.......G....A................T...C....	1	KX779738
Hap_211	...........C..G...T......T.A................T.............T...G...................................C....	1	KX779739
Hap_212	...........C..G...T......T.A........T........G..............A.....CG......T..................T.........	1	KX779740
Hap_213	...........C..G...T......T.A................TG......G..........C..CG.................T................C	2	KX779741
Hap_214	...........C..G...T......T.A................TG......G..........C..CG.................T.................	1	KX779742
Hap_215	...........................A................T..G..C....	1	KX779743
Hap_216	....G..........................T............................	1	KX779744
Hap_217	...............C...........A...............A................A.....................................C....	1	KX779745
Hap_218	.....C.....................A...............A..C....	1	KX779746
Hap_219	...........................A...C...........A..C....	1	KX779747
Hap_220	...........................A...............A..C....	8	KX779748
Hap_221	...........................A...............A..C.........C....	8	KX779749
Hap_222	...........................A..............GA..C....	1	KX779750
Hap_223	...........................A...............A..G...C....	1	KX779751
Hap_224	...........................A...............A...C..........C....	1	KX779752
Hap_225	.......C...................A.............A.A..C....	1	KX779753
Hap_226	...........................A...............A...T....C....	1	KX779754
Hap_227	....G.........G...........CA...............AT.......G.....................T.......................C....	1	KX779755
Hap_228	.....C.....C..G...T......T.A.......T.........G......G.............CG...............................A...	1	KX779756
Hap_229	.....C.....C..G...T......T.A............G....T......G...........G..G...............................A...	3	KX779757
Hap_230	....GC.....C..G...T......T.A............G....T......G...........G..G...............................A...	2	KX779758
Hap_231	.....C.....C..G...T........A............G....T......G...........G.....................G............A...	1	KX779759
Hap_232	..............G...........CA................T.......G....................CT.......................C....	1	KX779760
Hap_233	....G.........G...........CA........................G....................CT.......................C....	1	KX779761
Hap_234	....G.........G....C......CA................T...............A.............T.......................C....	1	KX779762
Hap_235	...........C..G............A...T..........C......	1	KX779763
Hap_236	...........................A...C................................G......................................	1	KX779764
Hap_237	...........................A............................C..	2	KX779765
Hap_238	..........................GA...........A................C..	1	KX779766
Hap_239	......C.......G..........T.A...CT.......................C....	1	KX779767
Hap_240	..............G.G.T.....GT.A.................G..G.............G....G......................T..T....C....	1	KX779768
Hap_241	..............G..........T.A...........A...	1	KX779769
Hap_242	..............G............A...........A....T.........................C...T.......................C....	1	KX779770
Hap_243	..............G............A...........A....T...C....	1	KX779771
Hap_244	..............G............A...........A....T..C......C....	1	KX779772
Hap_245	...........................A...........A....T...C....	2	KX779773
Hap_246	...........................A...............A.............................C.............................	1	KX779774
Hap_247	...........................A...............A..............................T............................	1	KX779775

H= Haplotype number, N= Haplotype frequency, AN= accession number.
