# Supplementary figures and images for: Genetic diversity and population structure of the primary malaria vector Anopheles sinensis (Diptera: Culicidae) in China inferred by cox1 gene
Source: Parasit Vectors. 2017 Feb 10;10:75. doi: 10.1186/s13071-017-2013-z (PMC5439230; doi:10.1186/s13071-017-2013-z)

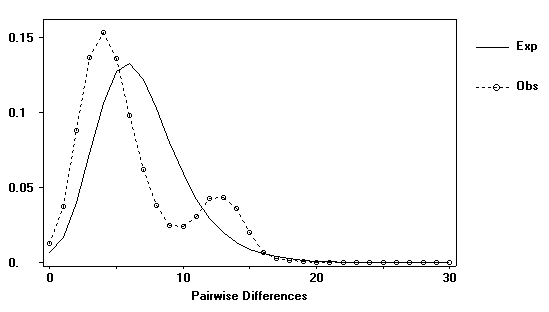

Supplement: Supplementary file 2 — Graphs of the mismatch distributions analysis for total populations of Anopheles sinensis using DnaSP 5.10. The X axis shows the observed distribution of pairwise nucleotide differences and the Y axis shows the frequencies. The dotted lines represent the observed frequency of pairwise differences, and the solid lines show the expected values under the sudden population expansion model. (TIF 8 kb) [file 13071_2017_2013_MOESM2_ESM.tif]
